# An Open-Source Platform for GIS Data Management and Analytics

**DOI:** 10.3390/s23083788

**Published:** 2023-04-07

**Authors:** Flavio Piccoli, Simone Giuseppe Locatelli, Raimondo Schettini, Paolo Napoletano

**Affiliations:** 1 Istituto Nazionale di Fisica Nucleare, 20126 Milano, Italy; 2 Dipartimento di Informatica, Sistemistica e Comunicazione, Università Milano-Bicocca, 20126 Milano, Italy

**Keywords:** Geographical Information Systems, remote sensing, multisource integration, soil characteristics estimation, precision agriculture

## Abstract

Precision agriculture has emerged as a promising approach to improve crop productivity and reduce the environmental impact. However, effective decision making in precision agriculture relies on accurate and timely data acquisition, management, and analysis. The collection of multisource and heterogeneous data for soil characteristics estimation is a critical component of precision agriculture, as it provides insights into key factors, such as soil nutrient levels, moisture content, and texture. To address these challenges, this work proposes a software platform that facilitates the collection, visualization, management, and analysis of soil data. The platform is designed to handle data from various sources, including proximity, airborne, and spaceborne data, to enable precision agriculture. The proposed software allows for the integration of new data, including data that can be collected directly on-board the acquisition device, and it also allows for the incorporation of custom predictive systems for soil digital mapping. The usability experiments conducted on the proposed software platform demonstrate that it is easy to use and effective. Overall, this work highlights the importance of decision support systems in the field of precision agriculture and the potential benefits of using such systems for soil data management and analysis.

## 1. Introduction

With the increase in population and the decrease in resources, sustainability is becoming an increasingly important issue and technological innovation can help to optimize all those processes that waste a lot of resources [1].

Agriculture is one of those sectors. In this context, the adoption of precision agriculture (PA) can dramatically contribute to improving sustainability [2]. Quoting the International Society of Precision Agriculture [3], “PA is a management strategy that gathers, processes and analyzes temporal, spatial and individual data and combines it with other information to support management decisions according to estimated variability for improved resource use efficiency, productivity, quality, profitability and sustainability of agricultural production”. Modern farms are sensorized environments that, thanks to the Internet of Things paradigm, enable the broader concept of Smart Agriculture [4,5,6,7].

In Smart Agriculture, data-driven Artificial Intelligence (AI) methods can help to support and/or automate management decisions [8]. Agricultural Decision Support Systems (DSSs) have been created in particular to support farmers in making effective ongoing and/or future decisions [8,9]. DSSs will guide effective agronomic practices to optimize the phases of mechanical processing, fertilization, weeding, irrigation, distribution of pesticides, and harvesting to a very high degree of detail by dosing the intensity according to the characteristics of the soil and the crops that have been measured and loaded into the system [10].

This significantly improves the entire process, thus reducing waste to a minimum and optimizing each intervention [11]. The use of data-driven technologies, however, require an expensive process of data collection and analysis. With the aim of avoiding mistakes, this process must follow strict acquisitions protocols and should be supported by an ad hoc software platform [12,13].

Furthermore, the scalability of DSSs on new areas (for which no samples are available) requires the use of AI-based predictive systems that are capable of performing digital soil mapping by estimating soil characteristics from remote sensors [14]. Specifically, it is possible to perform soil characteristic estimation from airborne sensors, such as drones or aerostatic balloons, and/or spaceborne sensors, such as satellites [15].

Data-driven AI methods require a large amount of data to be stored, managed, annotated, visualized, explored, and eventually edited [16]. Most importantly, the DSSs should provide an intuitive and effective graphical interface to permit result interpretability for human supervision and understanding [17,18]. To this end, Geographical Information Systems (GISs) are fundamental tools for the management, analysis, editing, and visualization of geospatial data [19,20]. There are free and open source software programs, such as QGIS (https://www.qgis.org/en/site/, accessed on 30 March 2023) as well as licensed-based software products, such as ArcGIS (https://www.arcgis.com/, accessed on 30 March 2023). Open source tools are very flexible, so they allow the creation of scientific and academic custom projects. Commercial tools are very robust to failures, making them very reliable in production environments.

In this work, we propose the Pignoletto web-platform, which addresses all the aforementioned topics for supporting data-driven precision agriculture. In particular, the proposed platform:

- Supports data collection and management from heterogeneous data sources;
- Provides all the tools for data visualization and decision making;
- Provides soil characteristics AI-based predictors;
- Provides a procedure to include custom predictors.

The platform and its components are open-source tools, leaving them easily modifiable and adaptable to other locations or specific needs.

The platform has been tested in the context of the Pignoletto Project (more info here https://www.pignolettomibinfn.it/, accessed on 30 March 2023), which is an Italian project about advanced tools for land monitoring. The data used are geophysical field measurements acquired in the region of Lombardy (northern of Italy) combined with airborne sensors (ionizing gamma radiation, optical hyperspectral, thermal multispectral), satellite information (PRISMA and Copernicus), and soil proximity measures.

The article is organized as follows: in Section 2, we describe the technological context; in Section 3, we describe the proposed platform; in Section 4, we present the selected use case; in Section 5, we present the results obtained through a survey on the usability of the platform; finally, in Section 6 and Section 7, we present our discussion and conclusions.

## 2. Scientific and Technological Context

Geographic Information Systems (GISs) have emerged as effective tools for the macro- and micro-level mapping of natural resources and artificial deployments; thus, their use extends to different sectors—from research to business [21]. There are several types of GIS software on the market, but the most widely used are QGIS [22] and ArcGIS [23]. GISs are powerful tools for collecting, storing, retrieving, transforming, and visualizing spatial data. Their ability to analyze and visualize agricultural environments and workflows has proven to be beneficial for the agricultural sector [24]. GIS technology is becoming an essential tool for combining different sources of data, such as data acquired by drones, airborne sensors, and satellites [25]. GIS tools are also playing an increasingly important role in precision agriculture, thus helping farmers to increase production, reduce costs, and manage their land more efficiently [26,27].

In the literature, GIS technology has been used in various scenarios. Frigerio et al. [28] proposed a methodology that exploits socio-economic factors to identify how different areas of the country can react to catastrophic natural events. Furthermore, GISs have proven to be valuable tools in preventing soil erosion and degradation processes [29].

Blanco et al. [30], instead, exploited the potential of GIS systems to monitor the use and waste of plastic in the Puglia (Italy) region; in particular, the tool developed allows stakeholders to quantify and localize areas characterized by intensive plastic production, thus allowing the study of the most suitable areas for collection centers.

Mancini et al. proposed methodology for the management of landslide hazard [31], while Ladisa et al. proposed methodology for the evaluation of desertification risks related to specific areas, as in [32].

Rossetto et al., on the other hand, integrated a toolset called FREEWAT [33] into the QGIS environment as a plugin to enable authorities to manage groundwater and surface-water resources using free and open-source software without the need for expensive licenses. The FREEWAT platform combines the capabilities of GIS geo-processing and post-processing tools for spatial data analysis with those of process-based simulation models. With FREEWAT, you can store large spatial datasets, manage and visualize data, and run several distributed modeling codes. FREEWAT can simulate hydrologic and transport processes and offers a database framework and visualization tools for hydrochemical analysis. The platform provides real-world case studies to demonstrate its applications.

Fegn et al. [34] developed GeoAPEXOL, a web GIS interface for the Agricultural Policy Environmental eXtender, which is able to evaluate the on-site nonpoint source (NPS) pollution status by applying Agricultural Policy Environmental eXtender (APEX) models on the whole-farm level or watershed scale, considering not only pollutants within the field boundaries but also outside, thus minimizing possible evaluation errors.

Zhang et al. [35] proposed an interoperable data service web application—the Crop Condition and Soil Moisture Analytics (Crop-CASMA) system—that facilitates the retrieval, analysis, visualization, and sharing of soil moisture data from remote-sensing measures.

DIVA-GIS [36] is a geographic information system software used for the creation of maps and the analysis of geographical data. The focus of this platform is the study of biodiversity. This tool also contains, for each country, basic information such as administrative areas, roads, land cover, etc.

## 3. Proposed Platform

### 3.1. Overview

The proposed platform is designed to handle geophysical field measures, airborne sensor measures (e.g., ionizing gamma radiation, optical hyperspectral, thermal multispectral), satellite information (e.g., PRISMA and Copernicus), and soil proximity measures. The platform provides tools for data visualization and decision making using AI-based predictors and custom predictors. The platform and its components are open-source tools, and it leaves the possibility of being easily modifiable and adaptable for other locations or specific needs.

**Figure 1:**
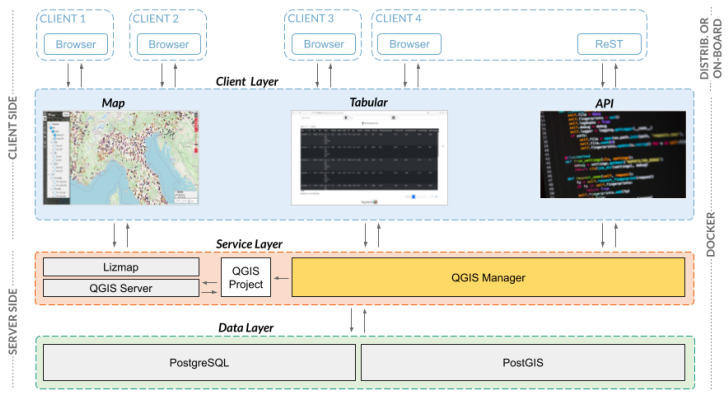
Architecture of the software. Clients can interact with the system through the Map, the Tabular, or the RESTful API. Our class QGISManager furnishes all the functions to perform CRUD operations on the platform database.

Figure 1 shows all components of the proposed Pignoletto platform and their interactions. The platform components can be grouped into server and client-side elements. The client-side is composed of several tools for interacting with data. The Map tool allows visual-based interaction with soil maps through a web browser, the Tabular tool allows interaction with data in a tabular format, and the API (Application Programming Interface) tool allows interaction with the platform through a computer program. The three interaction modalities are described in more detail, respectively, in Section 3.3, Section 3.4 and Section 3.5.

The server-side is composed of a service layer, which orchestrates the interactions between the clients and the data layer. Specifically, for the Map component, the service layer creates, at run time, a QGIS project and the corresponding Lizmap [37] configuration for the creation of the web interface. For the Tabular and API components, the service layer exposes proper functions for data uploading, querying, and editing. Data are stored in a PostgreSQL database with a CRUD-type storage engine (create, read, update, and delete). Please refer to Section 3.2 for further details. The use of QGIS and its utilities allows the sharing of platform geographical data through the Web Map Service (WMS) and the Web Feature Service (WFS), which are well-known standards in the GIS community [38].

The platform considers different types of users: (1) anonymous clients; (2) registered clients; (3) administrator. Anonymous users can only navigate the Map component, without any possibilities to edit the content. Registered users can interact with all the components of the client layer. They can add new data to the platform, but they can not delete existing data. Newly subscribed users must be manually approved by an administrator. The administrator has all the privileges to edit platform data.

### 3.2. Platform Database

**Figure 2:**
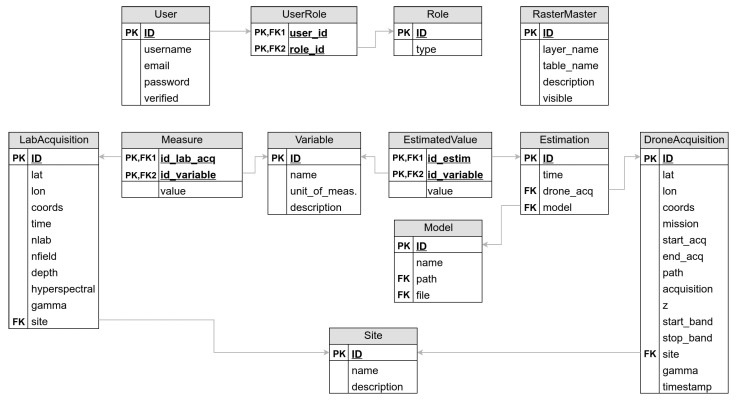
Database organization. It considers five aspects, the handling of users, the acquisitions made in the laboratory and with airborne sensors, the rasters, and finally the predictions made by predictive models on airborne-acquired data. The considered variables are not a static set, but they can easily change as the researchers focus on different characteristics of soil.

The platform handles two different types of land measures: (1) laboratory L measures and the remote (e.g., drone, satellite, etc.) acquisitions D. The former set can be collected in a controlled environment to support the training of deep-learning-based systems, while the latter set is composed of drone acquisitions. The database is designed to handle registered users, laboratory L and remote D (e.g., drone, satellite, etc.) acquisitions, as well as machine learning predictions and remote-sensing rasters. Figure 2 shows the logical organization of the platform database, with the main entities and relationships between them.

Let $s_{L}$ and $s_{D}$ be, respectively, two samples of L and D. $s_{L}$ and $s_{D}$ are located by means of geographic coordinates (latitude and longitude). They can belong to zero as well as to multiple sites. A site is a logical grouping of acquisitions, such as a sampling campaign or a field. Each $s_{L}$ consists of a hyperspectral signal associated with the measurement of one or more soil variables. This flexibility allows the set of soil properties considered to be modified at any time. For example, separate acquisition campaigns that consider different sets of variables can be modeled seamlessly in the system. These samples constitute the set of data used for the training of supervised predictors. Samples $s_{D}$ are not associated with soil properties. In fact, they represent specimens loaded at test time that require a prediction by an AI-based model. For this purpose, they are associated in the database with one or more estimates that in turn are associated with the model that made them in combination with the variables that were predicted by that model. This allows different variables to be predicted by multiple predictive models.

Each raster is represented through a pyramidal view in order to decrease the loading time. Each raster is therefore represented by a meta table that contains the information of each layer of the pyramid associated with the proper level of zoom. We also designed the Table RasterMaster to keep track of all the rasters in the system, with the possibility of disabling the visualization of a raster at runtime.

The platform database has been developed in PostgreSQL. To support georeferenced data, we enriched the suite by adopting PostGIS. This extension allows the use of geographic objects following the specifications from the Open Geospatial Consortium (OGC) (https://www.ogc.org/, accessed on 30 March 2023). OGC is a consortium of experts committed to improving access to geospatial or location information. Thousands of organizations worldwide rely on OGC Standards as the binding force for geospatial information interoperability, which is reflected in millions of lines of code.

### 3.3. The Map Component

The Map is displayed as a Web Map Service (WMS) application built using a custom version of the Lizmap API (https://github.com/3liz/lizmap-plugin, accessed on 30 March 2023) [37]. It is built on top of a QGIS server that displays the information contained in a QGIS project on a geographical map. It includes several Lizmap tools that allow the user to interact and explore the data. Table 1 describes these tools in detail. For instance, it is possible to filter data on the basis of their properties, to show the information about the collected data in a tabular way, etc. The Web Processing Service (WPS) component can be used to apply one of the available data processing algorithms to the selected samples. Later in the experimental section, we will show the application of the organic carbon map obtained through the inverse distance weighting (IDW) interpolation.

The QGIS project rendered through the Map is created by the service layer when the platform is started. Contextually, the service layer creates a Lizmap JSON configuration file for setting up the Lizmap parameters. Data inserted, modified, or deleted during the life cycle of the platform are immediately available (or hidden in the case of deletion) on the Map as soon as the operation is completed.

**Figure 3:**
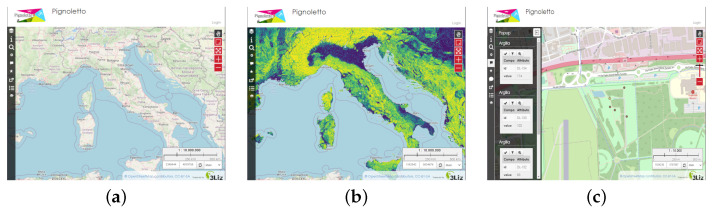
Main functionalities of the platform: (**a**) Initial view; (**b**) Raster layer selected; (**c**) Zoom on acquisitions.

Figure 3 shows three examples of interactions with the Map. In Figure 3a, it is possible to see the base map brought by OpenStreetMap [39]; in Figure 3b, the user has selected the visualization of a raster layer; in Figure 3c, the user zoomed on a field for observing the acquisitions.

### 3.4. The Tabular Component

**Figure 4:**
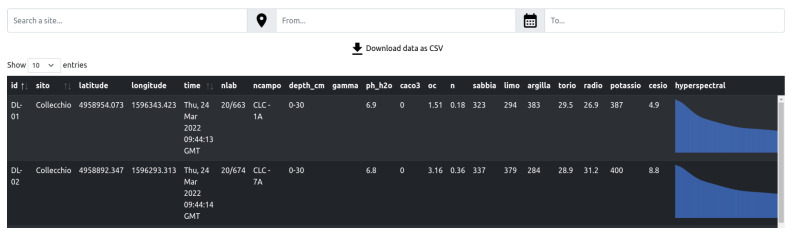
Closeup of the Tabular interface. Each sample is identified through an id and spatially located by its GPS coordinates. Each sample is characterized by its soil properties and by its hyperspectral signal, which were reported graphically for a better user experience.

The Tabular displays data in a tabular format. It is composed of two pages for handling, respectively, the acquisitions L and D. Figure 4 shows an excerpt of L samples. In this context, each lab sample is characterized by a numerical id, the position expressed in latitude and longitude, and all the chemical properties related to it. To make the visualization of the samples more effective, the last column includes a graphical plot of the hyperspectral signal instead of the raw values.

While the Map is visible without authentication, the Tabular is accessible only to registered users. Among them, two types are identified: the normal users, who can view, filter, sort, and download data, and the administrator who can go further and modify the data stored in the dataset. In particular, they can upload one or more samples of L and D at once through a CSV file and upload a zipped archive of images or new predictive models that can be used later for estimating variables.

The Tabular is a Flask-based web application developed in Python that queries the data through SQLAlchemy. Information is gathered through the browser and thus rendered with HTML/CSS/JS. Two external libraries are used: Datatable (https://datatables.net/, accessed on 30 March 2023) for rendering the tables and Peity (http://benpickles.github.io/peity/, accessed on 30 March 2023) for plotting the spectral signals.

### 3.5. API

The platform API allows the uploading, querying, and editing of D samples. New samples can be loaded in real-time from a software running on the acquisition device. Two modalities have been identified: a single and a massive upload. The former can be performed using the HTTP protocol and a JSON-formatted request, while the latter through a CSV file. Each line of the file is a sample that must be inserted into the system.

For convenience, the API is included in the same Flask web app of the Tabular as the two elements share several functions. It has been implemented following the REpresentational State Transfer (REST) style, whose principle fits nicely with the HTTP protocol used for communication.

The JSON Web Token (JWT) open standard is used to improve the security of the system. In the first place, the user authenticates the system and receives, through an HTTP response, a token that represents its identity. Further on, this token enables the use of API capabilities.

**Figure 5:**
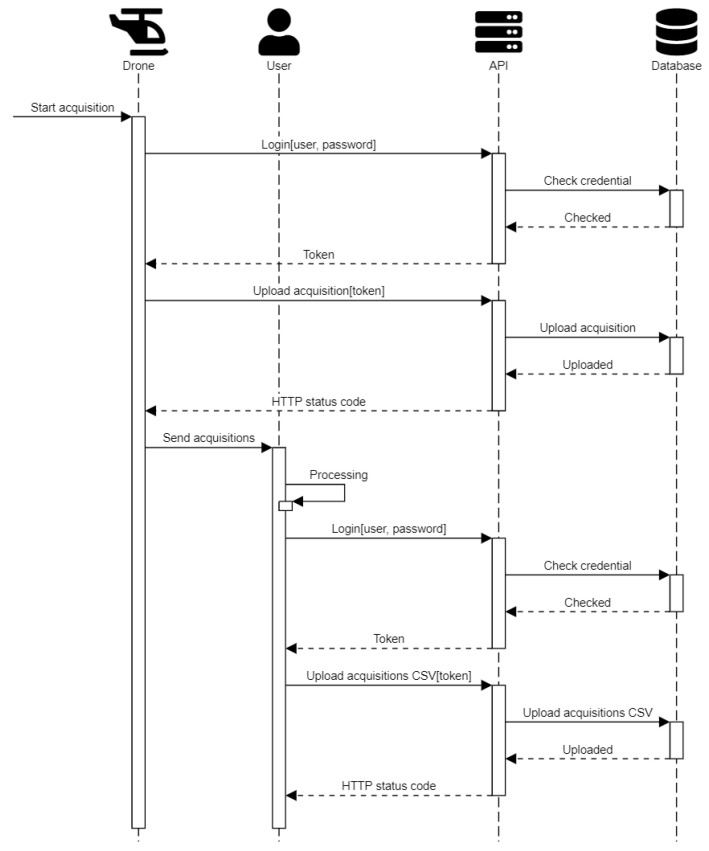
High-level flow of API’s execution. On-board devices and users can interact programmatically with the system through the API. After the logging procedure performed through the token, a single or massive upload of acquisitions can be performed.

An example of the API usage is presented in Figure 5. In this example, we show the communication flow between the user—or an acquisition device—and the platform. In the first place, the user requires the authorization to interact with the database through the service layer, and in second place, it performs a single and massive data uploading.

## 4. Use Case

The proposed platform has been tested within the Pignoletto (https://www.pignolettomibinfn.it/il-progetto, accessed on 6 March 2023) project, which aims to target the specific agricultural needs of the Lombardy region, including the automatic creation of soil characteristic maps of the agricultural lands on the basis of proximity, as well as remote and hybrid chemical and spectral measures. This will support local farmers in adapting their practice to crop management. Better crop management is a key objective of precision agriculture, and it involves a range of techniques, such as precision planting, irrigation, and fertilization. By using sensors, satellite imagery, and other advanced tools, farmers can analyze various factors such as soil moisture, nutrient levels, and crop growth patterns in real-time. This information helps farmers to make more informed decisions about crop management, enabling them to apply resources such as water and fertilizer more precisely and at the right time, leading to improved crop yields and reduced environmental impact.

The project considers two different types of land measures: (1) laboratory L measures and drone acquisitions D. The former set is collected in a controlled environment to support the training of a deep-learning-based system *M* able to estimate soil properties *p* from the spectral information *h*, i.e., $M\left(h\right)=\hat{p}$. To this aim, each sample (h,p)∈L is composed of the hyperspectral signal *h* together with the soil properties *p* measured by an expert. The latter set is composed of drone acquisitions that will be loaded at test time. These acquisitions are acquired in the wild. Once they are loaded in the system, one or more predictive systems *M* will perform an estimation of the soil properties $\hat{p}$ associated with this signal.

## 5. Experimental Results

Two usability tests were performed to evaluate the ease of use of the Map and the Tabular components. The first one is a task-driven survey (TDS) containing the 11 questions showed in Table A1. Each question asks to perform a task and assess the easiness of doing it. In this case, the higher the score is, the better the goodness is. The second one is the System Usability Scale (SUS) [40]. For odd questions, the higher, the better. On the contrary, for even questions, the lower, the better.

**Figure 6:**
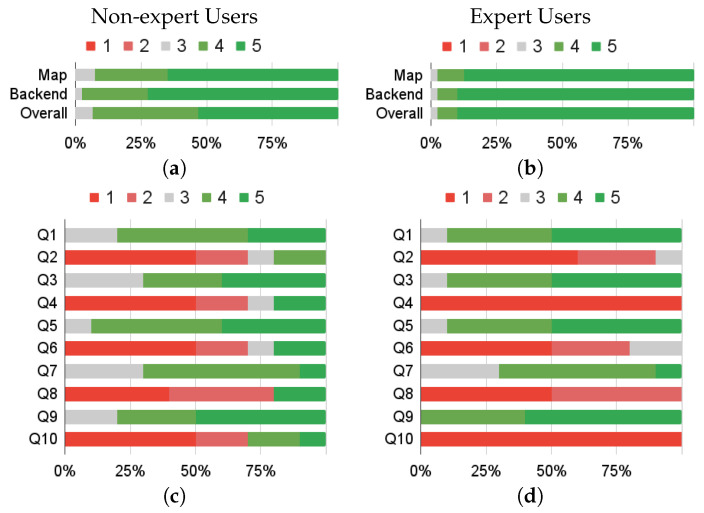
The first row represents the results of task-driven assessment (TDS) for non-expert (**a**) and expert (**b**) users. The second row shows the SUS assessment for non-expert (**c**) and expert (**d**) users. For TDS and odd questions of SUS, the higher, the better. For even questions of SUS, the lower, the better.

In total, 20 users were involved in the usability assessment, and 10 of them had expertise in GIS-based systems. The first row in Figure 6 represents the results of the task-driven tests, while the second row represents those of the SUS test divided according to user expertise. All evaluations demonstrated that the Map and the Tabular are easy to use, and the learning curve is low both for non-expert and expert users. From the analysis of the replies to question 3 of TDS, it emerged that users prefer more levels of zoom, which has been corrected after the evaluation. As expected, from question 4 and 10 of the SUS, it emerges that expert users were more confident in using this tool. Specifically, none of the expert users expressed the need for technical support, nor the need for training before using the tool.

All experiments were timed. Normal users took an average of 10 min, while experienced users took 5 min. Again, this confirms that previous experiences with GIS tools favor the use of the proposed tool. Interestingly, all non-expert users were also able to complete the tasks, and their times are still low.

**Figure 7:**
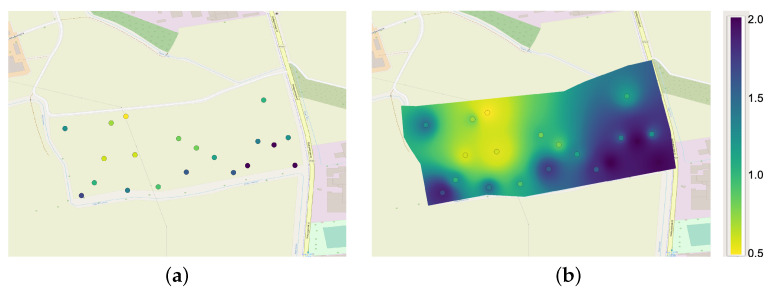
Example of Organic Carbon visualization: (**a**) soil sample; (**b**) soil mapping obtained using IDW interpolation.

To better demonstrate the potential of the WPS component of the platform, in Figure 7, we show the organic carbon map obtained through the inverse distance weighting (IDW (https://docs.qgis.org/3.22/de/docs/user_manual/processing_algs/qgis/interpolation.html, accessed on 6 March 2023)) interpolation. This map is obtained from 20 laboratory samples of organic carbon taken from 20 ha of soil in the surrounding of the city of Lodi. (Lodi is a city in the southern Milan (Italy). The center of the area under analysis is at the following coordinates: lat 45.29167066234336 and long 9.50092884268406.) The digital map obtained is at a resolution of about 0.5 m per pixel.

### Software Availability

All technologies used for the creation of the platform are free and open source. To make the system usable in any context and to maximize reproducibility, a Docker [41] environment containing all the necessary software and data samples was created. You can find the GitHub repository of the proposed platform (https://github.com/SimoLoca/Pignoletto_platform, accessed on 6 March 2023).

The server is hosted on an Ubuntu 20.04 machine composed of an ASRock Z68 Extreme3 Gen3 motherboard and an Intel(R) Core(TM) i5-2500K CPU @ 3.30GHz. All code is included in a Docker image containing all the dependencies. The web server used is NGINX. A DNS record has been bound to the server IP to make the platform easily accessible through the URL http://pignoletto.lombardia.it, accessed on 6 March 2023.

Table 2 describes in detail all the attributes related to the presented software.

## 6. Discussion

In this section, we analyze commonalities and differences among the proposed tool and the platforms that compose the state of the art for digital soil mapping. The analysis was conducted by considering four key aspects that characterize a GIS system.

Focus: FREEWAT [33] has been designed for managing ground- and surface-water resources. The focus is then applied to modeling the water flow through hydrological models. GeoAPEXOL [34] has been crafted with the goal of evaluating nonpoint source pollution (NPS), e.g., pollution coming from contaminants that end up on the ground or from human activity. To this aim, the platform offers several field and small watershed simulations for predicting NPS. Crop-CASMA [35] has been created for studying biodiversity through remote sensing. It contains remotely sensed geospatial soil moisture and vegetation index data. DIVA-GIS [36] is a generic tool for the creation of maps. The proposed method differs from state-of-art methods as it proposes several tools for supporting the manual annotation of ground points (e.g., tabular, map-based, and csv-based). It also allows the creation of digital soil maps and second-level analyses. To prove its effectiveness, the proposed platform has been used to collect and analyze a library of soil properties relative to the Lombardy region in Italy.
End-user type: The definition and development of user processes in a GIS-based platform is guided by the skills expected of the end user. In this context, FREEWAT [33], GeoAPEXOL [34], and Crop-CASMA [35] are intended for experienced users, such as researchers. DIVA-GIS [36] and the proposed platform are designed for both expert and non-expert users. In the case of the Pignoletto platform, this is also possible due to the fact that the platform is data-agnostic, that is, the ground-points to be inspected can represent any kind of data or information.
Technological context: The technology used in the development of a platform influences its reach and spread. FREEWAT [33] is a QGIS plugin. This limits its effectiveness only to QGIS users. DIVA-GIS [36] has been developed for Windows and Mac OSX only. In addition, the tool is becoming obsolete as the last update was performed in 2011. GeoAPEXOL [34], Crop-CASMA [35], and the proposed platform are delivered through a web page, allowing their use on any platform through a web browser.
Integrability: Platforms can be closed stand-alone products, or they can allow the exposition of the data gathered and inferred through the offered functionalities. FREEWAT [33] is fully integrated in QGIS, and thus, its interoperability is granted by the QGIS environment. DIVA-GIS [36] and GeoAPEXOL [34] are closed environments that do not allow a direct data exposition. Crop-CASMA [35] and the proposed platform implement the GIS WMS standard. This allows the exposition and the direct integration of the data under analysis in other GIS-based platforms. In this conception, the proposed platform can become a data collection and inspection module in a broader pipeline.

## 7. Conclusions

In this paper, we presented a new platform for handling and processing airborne and proximity data. It is also capable of integrating predictive models and their relative predictions.

The presented tool intends to support all stakeholders related to the agricultural sector in adopting the best practices of PA with the aim of improving the entire agricultural production chain. Although it was initially published to support the specific needs of the Lombardy region, the platform is easily extendable to other locations with different needs, thus making it a software exploitable for any purpose. The usability assessment that we performed in Section 5 showed that the presented system is easy to use, and the learning curve is low both for users with and without previous experience in GIS systems.

Those factors, in combination with the technical gain offered by the platform, suggest that the presented system can bring a noticeable impact on the agricultural sector thanks to its effective utility and ease of use. The platform we presented in this paper can be easily customized to meet the specific needs of different regions, making it a valuable tool for promoting sustainable agriculture around the world. By using the platform to optimize their agricultural practices, farmers can also reduce the overall carbon footprint of their operations, contributing to global efforts to mitigate climate change.

Moreover, the presented tool can benefit society in several ways. Firstly, it can improve the quality and quantity of agricultural production, leading to increased food security and availability, which is especially crucial in areas with high levels of food insecurity. Secondly, by reducing the use of agricultural inputs and minimizing the environmental impact of farming practices, the platform can contribute to the sustainable development of rural areas and protect the health and well-being of people living in these areas.

In summary, the presented platform is a valuable tool for promoting sustainable agriculture, reducing the environmental impact of farming practices, and contributing to global efforts to mitigate climate change. By optimizing agricultural practices and improving the efficiency of the entire production chain, the platform can benefit farmers, rural communities, and society as a whole.

## Figures and Tables

**Table 1:** Utilities included in the web-map.

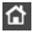	**Home:** brings the user back to the project selection page.
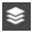	**Layers:** to control the visibility of the layers on the map
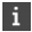	**Information:** gives information about the project
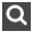	**Filter:** lets the user filter data based on their properties.
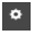	**WPS:** for choosing a model (or algorithm) uploaded and extract useful informations.
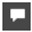	**Popup:** it will show information about the data clicked by the user.
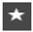	**Selection:** enables the drawing on the map.
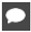	**Tooltip:** It will highlight data of the layer under inspection that lies under the cursor.
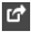	**Permalink:** enables the sharing of the link.
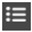	**Data:** shows informations about collected data in a tabular way.
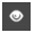	**WPSResults:** shows the results of the algorithms used.

**Table 2:** Details of the software.

Attribute	Value
Name of the proposed software	Pignoletto platform
Availability	https://github.com/SimoLoca/Pignoletto_platform, accessed on 6 March 2023
Developers	Locatelli, Piccoli, Napoletano, Schettini
Contact	flavio.piccoli@unimib.it
Licence	MIT License
Hardware required	2+ GHz processor. 4+ GB of RAM
Software required	Python 3.x, Docker
Program Language	Python 3.x
Program size	26.3 MB

## Data Availability

Data sharing is not applicable to this article.

## Appendix A

### Questions of Task-Driven Assessment

Table A1 contains the questions posed to the users in the TDS test.

**Table A1:** Task-driven questions.

Map	Display the acquisitions relative to the Argilla variable under the group lab_acquisition. Zoom on a point and click on it to view its value. How hard is this task?
	Open the tabular visualization (“Dati” tool on the left sidebar) and click on the button relative to the Argilla acquisitions to open the table. Locate the sample DL-06 in the table and click the lens icon to view the corresponding point on the map. Click on the found point to view the relative info on the left panel.
	Zoom out to have all the acquisitions (points) on the screen visible. Search all the samples that contain a level of Argilla between 200 and 400 (use the “Filtro” tool on the left sidebar).
	Display the raster slope (it can take some time to load).
Tabular	Download the dataset about Laboratory acquisition.
	Filter the samples that have been acquired in Lodi.
	Sort the “sito” column on the Laboratory acquisitions in descending order.
	Go to the Drone acquisition page and try to filter the samples that have been acquired from 5 April 2022 to 14 May 2022.
General	How user-friendly is Pignoletto platform map interface?
	How user-friendly is Pignoletto platform tabular interface?
	Do you think this application can provide valuable support for precision agriculture?
